# Effect of Improved access to Antiretroviral Therapy on clinical characteristics of patients enrolled in the HIV care and treatment clinic, at Muhimbili National Hospital (MNH), Dar es Salaam, Tanzania

**DOI:** 10.1186/1471-2458-10-291

**Published:** 2010-05-28

**Authors:** Sabina F Mugusi, Julius C Mwita, Joel M Francis, Said Aboud, Muhammad Bakari, Eric A Aris, Andrew B Swai, Ferdinand M Mugusi, Kisali Pallangyo, Eric Sandstrom

**Affiliations:** 1 Department of Internal Medicine, Muhimbili National Hospital, Dar es Salaam Tanzania; 2 Department of Internal Medicine, Muhimbili University of Health and Allied Sciences, Dar es Salaam Tanzania; 3 Department of Microbiology, Muhimbili University of Health and Allied Sciences, Dar es Salaam Tanzania; 4 Department of Infectious Disease, Karolinska Institute, Stockholm Sweden

## Abstract

**Background:**

Sub-Saharan Africa has been severely affected by the HIV and AIDS pandemic. Global efforts at improving care and treatment has included scaling up use of antiretroviral therapy (ART). In Tanzania, HIV care and treatment program, including the provision of free ART started in 2004 with a pilot program at Muhimbili National Hospital in Dar es Salaam. This study describes the socio-demographic and clinical features of patients enrolled at the care and treatment clinic at MNH, Dar es Salaam, Tanzania.

**Methods:**

A cross-sectional study looking at baseline characteristics of patients enrolled at the HIV clinic at MNH between June 2004 - Dec 2005 compared to those enrolled between 2006 and September 2008.

**Results:**

Of all enrolled patients, 2408 (58.5%) were used for analysis. More females than males were attending the clinic. Their baseline median CD4 cell count was low (136 cells/μl) with 65.7% having below 200 cells/μl. Females had higher CD4 cell counts (150 cells/μl) than males (109 cells/μl) p < 0.001). The most common presenting features were skin rash and/or itching (51.6%); progressive weight loss (32.7%) and fever (23.4). Patients enrolled earlier at the clinic (2004-5) were significantly more symptomatic and had significantly lower CD4 cell count (127 cells/μl) compared to CD4 of 167 cells/μl in those seen later (2006-8) (p < 0.001).

**Conclusion:**

Patients enrolled to the MNH HIV clinic were predominantly females, and presented with advanced immune-deficiency. Improved access to HIV care and treatment services seems to be associated with patients' early presentation to the clinics in the course of HIV disease.

## Background

HIV infection is a global pandemic. By the end of 2007 it was estimated that about 33.2 million people were living with HIV in the world. Among these, 22.5 million (68%) are in sub-Saharan Africa [1].

Although the new UNAIDS and WHO reports show leveling off of the HIV global prevalence as well as a decrease in the number of new infections, HIV/AIDS is still a problem that needs attention. It is thought that the noted downward trend is in part a result of the impact of HIV programmes [1]. The prevalence of HIV in Tanzania during the year 2007 was 7% (11% in urban and 5% in rural areas) [1]. In response to the global efforts at improving care and treatment, the Tanzania government in collaboration with various partners started the HIV care and treatment in 2004 that included the provision of free antiretroviral drugs [2]. Despite the generally known fact that HIV-1 infection invariably leads to AIDS and death, it is increasingly being reported that certain HIV-1 infected individuals remain healthy and free from AIDS for as long as 15 yrs or more. A study conducted in Dar es Salaam, Tanzania showed that with a definition of maintaining a CD4+ cell slope ≤ -10 (loss of 10 or less cells per year) 26% were long term slow progressors [3].

The program started with a pilot phase conducted at the Muhimbili National Hospital (MNH), a tertiary referral hospital in Dar es Salaam Tanzania. Since then care and treatment expanded to cover the entire country. Patients are enrolled and cared for in HIV clinics according to the set National guidelines[4]. Easy access to care and treatment, may have changed characteristics of HIV infected persons seeking treatment in treatment centers. This study aimed at describing changing social demographic and clinical characteristics of patients enrolled at the Muhimbili National Hospital HIV clinic as the ART program has developed.

## Methods

The HIV care and treatment clinic (CTC) of MNH, in Dar es Salaam, Tanzania was set up in June 2004. The clinic was part of the National HIV care program that was started countrywide to provide care and treatment including provision of free ARV's. Patients' clinical information at the clinic was monitored using a clinic visit form. This information was and is still being regularly entered into the computer. Data of patients enrolled between June 2004 and September 2008 was used for analysis. Eligible patients for this analysis were those aged 18 years and above. The clinic enrolled HIV positive patients referred from voluntary testing and counseling centers as well as hospitals in and around Dar es Salaam. At enrollment a structured first-visit form was used to collect patients' social demographic and clinical information, physical findings and anthropometric measurements. Opportunistic infections were diagnosed on the basis of standard clinical definitions and individual laboratory investigations. CD4 T-lymphocyte counts were determined using FACS Count System (Becton Dickinson, San Jose, CA, USA). HIV disease severity was categorized using the WHO HIV disease staging whereby stage I and II was categorized as mild disease, stage III as moderate disease and stage IV was categorized as severe disease. Immune suppression as indicated by CD4 T-cell counts was divided into < 100 cells/μL, 100-200 cells/μL and > 200 cells/μL.

### Ethical Issues

The study received ethical clearance by the Muhimbili University of Health and Allied Sciences (MUHAS) and permission to conduct the study was granted by Muhimbili National Hospital (MNH).

### Statistical analysis

Data were extracted from the hospital's Access data base and analyzed using a statistical package for social sciences (SPSS for windows, version 12.0). Means (Standard deviations) and medians (Interquartile Ranges) were used to describe the distributions of continuous variables. Percentages were used to describe categorical variables. Comparisons of categorical data were performed with the use of Pearson's chi-square test. For continuous data a Student *t*-test was used to compare means.

## Results

Between June 2004 and September 2008 the hospital enrolled 4108 patients to its HIV clinic but only 2408 patients had complete documentation on the database and were used for this analysis. Incomplete data for this analysis included all individuals whose identification was in doubt, i.e. names, age and sex were not indicated or were actually in doubt. Also incomplete data included all those who at baseline had no clinical information in their records and had no baseline CD4 counts or did not have clinical HIV staging. Individuals missing any one of these important variables were excluded. Patients who were excluded had similar social and demographic characteristics to those finally analysed. (Table 1)

**Table 1:** Social demographic characteristics and HIV stage of the study population (n = 4108).

Baseline characteristics		Excluded N = 1700 (41.4%)		Included N = 2408** (58.6%)	p-value
Median Age (IQR)		906*	37 (16)	37 (12)	0.1173
Age groups	18-30	906*	183 (20.2)	458 (19.0)	0.8718
	31-40		387 (42.7)	1054 (43.8)	
	41-50		224 (24.7)	604 (25.1%)	
	> 50		112 (12.3)	292 (12.1)	
Sex	Males	1342*	424 (31.6)	741 (30.8)	0.6019
	Females		918 (68.4)	1667 (69.2)	
Marital status	Married	786*	358 (45.5)	1133 (47.1)	0.7583
	Never married		284 (36.1)	842 (35.0)	
	Separated/widowed		144 (18.3)	433 (18.0)	
HIV stage	I and II	426*	288 (67.6)	1642 (68.1)	0.7332
	III		103 (24.2)	548 (22.8)	
	IV		35 (8.2)	218 (9.1)	

*Number of individuals with available data in the excluded group

** Number of individuals with analysed data in the table

Table 2 summarizes the baseline characteristics of the studied patients by recruitment period. The overall median age at enrollment was 37 years, with males being significantly older (p 0.003). Most patients (62.7%) were below 40 years of age, with twice as many females as males (ratio 2.2:1). Most patients (47.1%) were married, and this was true among both male and female patients. The mean body mass index (BMI) of the studied patients was 22.8 ± 4.6 kg/m2.

**Table 2:** Baseline Characteristics of enrolled patients at the HIV clinic at MNH 2004 to 2008 (N = 2408).

Characteristics	All Patients (N = 2408)	Enrolled 2004-5 (N = 1551)	Enrolled2006-8 (N = 857)	P Value
***Sex***				
Male	741(30.8)	489(31.5)	252(29.4)	
Female	1667(69.2)	1062(68.5)	605(70.6)	0.280

***Marital status***				
Married	1133(47.1)	654(42.2)	479(55.9)	
Never married	842(35.0)	586(37.8)	256(29.9)	**< 0.001**
Separated/widowed	433(18.0)	311(20.1)	122(14.2)	

Median age (IQR)*	37(12)	38(12)	37(12)	**< 0.003**

Mean BMI, kg/m^2 ^(SD)	22.8 ± 4.6	22.8 ± 4.6	22.9 ± 4.7	0.58

Median CD4 count, cells/μl (IQR)	136(203)	127(174)	167(256)	**< 0.001**

***WHO stages***				

Stage I&II	1642 (68.1)	968 (62.4)	674 (78.6)	**< 0.001**
Stage III	548 (22.8)	407 (26.2)	141 (16.5)	
Stage IV	218 (9.1)	176 (11.3)	42 (4.9)	

***Past medical history***				

Herpes Zoster	474(19.7)	358(14.9)	116(4.8)	**< 0.001**

Tuberculosis	495(20.6)	397(16.5)	98(4.1)	**< 0.001**

***Type of HIV treatments at enrollment***				

No treatment	1942(80.6)	1089(70.2)	853(99.5)	**< 0.01**

ARVs	401(16.7)	398(25.7)	3(0.4)	**< 0.01**

Spiritual	19(0.8)	19(1.2)	(0)	**< 0.01**

Traditional	46(1.9)	45(2.9)	1(0.1)	**< 0.01**

***Symptoms***				

Progressive Wt loss	787(32.7)	471(30.4)	316(36.9)	**0.001**

Skin rash	623(25.9)	438(28.2)	185(21.6)	**< 0.001**

Fever	564(23.4)	306 (19.7)	258(30.1)	**< 0.001**

Productive cough	463(19.2)	288(18.6)	175(20.4)	0.27

Numbness	405(16.8)	269(17.3)	136(15.9)	0.354

Night sweat	358(14.9)	191 (12.3)	167 (19.5)	**< 0.001**

Abdominal pain	312(13.0)	171(11)	141(16.5)	**< 0.001**

Dry cough	246(10.2)	165(10.6)	81(9.5)	0.357

Anorexia	202(8.4)	142(9.2)	60(7)	0.178

Vomiting	188(7.8)	103(6.6)	85(9.9)	**0.003**

Nausea	182(7.6)	106(6.8)	76(8.9)	0.149

Genital lesions	155(6.4)	101(6.5)	54(6.3)	0.840

Diarrhoea	127(5.3)	29(1.9)	98(11.4)	**< 0.001**

Odynophagia	93(3.9)	66(4.3)	27(3.2)	0.178

*IQR is Inter quartile range

At presentation to the clinic patients who were enrolled earlier (2004-2005) 62.4% were WHO stages I and II, 26.2% and 11.3% were WHO stages III and IV respectively. For patients who were enrolled later (2006-2008) 78.6% were WHO stages I&II, with 16.5% and 4.9% of them being WHO stages III and IV respectively. These findings were found to be statistically significant (p = < 0.001). Patients had an overall median CD4+ T lymphocyte count of 136 cells/μl (IQR 203). Two thirds (65.7%) of all patients had CD4+ T lymphocyte counts below 200 cells/μl. Of those with CD4+ T lymphocyte counts below 200 cells/μl, more than half (61.5%) had CD4+ T lymphocyte counts below 100 cells. The median CD4+ T lymphocyte count for females (150 cells/μl) (IQR 212) was significantly higher than that of males (109 cells/μl) (IQR 174), p < 0.001.

Patients enrolled during the first year of the program (2004-5) had a significantly lower median CD4+ T lymphocyte count of 127 cells/μl (IQR 174) compared to that of 167 cells/μl (IQR 256) among those enrolled in 2006-8, (p < 0.001). Many, (26%) were ART experienced during the first period, while almost none had received ART during the latter period. (*p *= 0.01).

The common clinical presentations among patients at the time of enrollment are shown in Table 2. The commonest presenting complaints were skin rash and/or itching (51.6%), progressive weight loss (32.7%), fever (23.4%) and general body weakness (20.7%). Pruritic purpura eruption, hair changes (described as silky hair) and oral candidiasis were the commonest documented clinical signs (Table 2). Weight loss, fever, abdominal pain were the commonly reported symptoms among patients enrolled in 2006-8 while numbness, lymphadenopathy and Seborrhoeic dermatitis were significantly common among patients enrolled in 2004-5.

In the past medical history patients reported to have suffered from Herpes zoster (19.7%) and Tuberculosis (20.6%) over the past 5 years. Those enrolled earlier reported to have suffered from Herpes zoster (14.9%) and Tuberculosis (16.5%) significantly higher than those who were enrolled later, 4.8% and 4.1% respectively (p = < 0.001).

### Common clinical symptoms and signs in relation to CD4 counts

As expected, at enrolment patients whose CD4+ T lymphocyte counts were low were more symptomatic than those with relatively higher counts. Furthermore, the frequency of symptoms was noted to significantly decrease with increasing CD4 counts. However, numbness, parasthesia, seborrhoeic dermatitis, Kaposi's sarcoma and lymphadenopathy were not related to patients' level of immunosuppression (Table 3).

**Table 3:** Common Clinical Symptoms and Signs by CD4 T-cell Counts among HIV-infected Patients (N = 2408).

Clinical features	CD4 T-cell Counts						
	< 100 (n = 974)		100-200 (n = 609)		> 200 (n = 825)		P-value
	N	%	N	%	N	%	
Wt Loss	418	42.9	170	27.9	199	24.1	**< 0.001**
Skin itching	307	31.5	157	25.8	156	18.9	**< 0.001**
Fever	288	29.6	121	19.9	155	18.8	**< 0.001**
General body weakness	273	28.0	111	18.2	114	13.8	**< 0.001**
Productive cough	251	25.8	90	14.8	122	14.8	**< 0.001**
Sweat	202	20.7	77	12.6	79	9.6	**< 0.001**
Numbness	185	19.0	104	17.1	116	14.1	**0.02**
Nausea/Vomiting	108	11.1	35	5.7	39	4.7	**< 0.001**
Dry cough	115	11.8	69	11.3	62	7.5	**0.006**
Paraesthesia	99	10.2	60	9.9	60	7.3	0.079
Diarrhea	76	7.8	23	3.8	20	3.4	**< 0.001**
Dysphagia	68	7.0	17	2.8	35	4.2	**< 0.001**
Hair changes	184	18.9	62	10.2	39	4.7	**< 0.001**
Oral Candidiasis	165	16.9	42	6.9	42	5.1	**< 0.001**
Pallor	114	11.7	42	6.9	35	3.2	**< 0.001**
Chest Crackles	54	5.5	17	2.8	19	2.3	**< 0.001**
Seborrheic dermatitis	52	5.3	27	4.4	18	2.2	**0.003**
Angular cheilitis	45	4.6	17	2.8	14	1.7	**< 0.001**
Kaposi's sarcoma	40	4.1	12	2.0	20	2.4	**0.026**

## Discussion

This study looked at the effect of easy access to antiretroviral therapy on the clinical characteristics of patients attending the HIV clinic at Muhimbili National Hospital. The clinical characteristics of patients seen during the early stages of the program (2004-2005) were compared with those seen in patients who presented later during the program (2006-2008) when access to antiretroviral therapy was wide spread in Tanzania and more so in the city of Dar es Salaam where the study took place. Most patients who reported to the clinic during the study period (2004 to 2008) had relatively severe immunosuppresion, this is indicated by their low median CD4 cell count at presentation (136 cells/μl [IQR 203]). Although patients who were enrolled to the clinic had severe immunosuppression, those enrolled during the first years of the program were found to be even more immuno-compromised as compared to those enrolled later. This was also true with clinical presentation, a lot more patients were symptomatic at the beginning of the program (2004-2005) compared to the period 2006-2008. This difference in immune status and clinical characteristics at the two periods can be explained by easy access of antiretroviral drugs which could have increased awareness and reduced stigma of the disease thereby leading to early presentation to HIV clinics.

Before the National care and treatment programs (2004) access to ARV's was very limited. Prior to the ART care and treatment program, some patients who came to the clinic were not ART naïve as those who could afford to buy their own ARV's did so. However due to the high cost, the experience at our clinic showed that there was poor adherence to proper ART. With the start of the care and treatment program, ARV's were provided free and therefore a larger proportion of people who came to the HIV clinic were ART naïve. Despite the free antiretroviral drugs a number of patients still do acquire their drugs from private pharmacies. Although drugs in these private pharmacies are a lot cheaper than before 2004, they are still expensive for most of the patients in Tanzania. A number of patients having failed to buy drugs would then come back to public HIV clinics. The absence and difficulty of access to appropriate treatment early in the program sometimes led to patients seeking alternative treatments. Some patients in this study (2.7%) used other forms of treatment for their illnesses including traditional medicines and/or spiritual healing. The absence or inaccessibility to modern medicine increases the proportion of patients using traditional or alternative medicines. With increased access to ART, there was a reduction of patients who had previously used traditional or alternative medicine as seen in patients who were enrolled in 2006-2008.

In this study males at presentation were more immunocompromised than females. The same finding was reported in a study done in Kenyan HIV clinics [5], though patients at MNH presented with even more severe immunosupression. The reason why males presented with lower CD4 cell counts was not clear from this study, however we can only speculate that men present late to the clinic because of stigma which is probably higher among men compared to women. The other reason could be the difference in health seeking behavior of men compared to that of women. It was found that females formed the majority of enrolled patients at the clinic. This finding is in agreement with other studies that found more female enrollment to the clinics than men [4]. This was true for both early and later in the program.

The results of this study indicate that young people below the age of 40 years accounted for the majority of clinic attendees. This is in keeping with earlier reported national and global findings on the age group of patients most affected with HIV [1,6,7].

It is apparent from this study that the time at presentation to the clinic for HIV care and treatment influences the clinical spectrum of symptoms and signs seen at the initial evaluation. Most patients find out their serostatus after having suffered from a HIV related disease that would have prompted a HIV test. VCT that is mainly performed in healthy individuals may not necessarily lead to a patient to seek medical care. In this study many patients had one or more symptoms at baseline. Indeed the number of symptoms at enrollment was found to be linearly inversely related to the level of CD4+ T lymphocyte counts. Similar findings have been reported from other studies as well [4,6].

About one fifth of patients reported to have suffered from tuberculosis or herpes zoster over the past 5 years prior to enrollment; this finding is similar to what has been found from other studies in Africa [4,8,9]. The history of having suffered from tuberculosis or herpes zoster was reported more frequently in those enrolled early in the program; this may be an indication of patients being more symptomatic among those who presented early in the program as compared to those who presented later. Most opportunistic infections reflect the available pathogens in the environment. Tuberculosis is reported to be the most common infectious disease from a single pathogen and it is highly prevalent in sub Sahara Africa and hence its frequent finding in HIV infected patients. The fact that Tuberculosis occurs even in patients with relatively good immune status will frequently be reported in the patients past medical history [10-12]. This could mean that easy access to ART makes patients come forward early in the HIV disease progression before they develop common opportunistic infections like tuberculosis and herpes zoster. Of note also is the fact that dermatological disorders were a very common presentation in patients in keeping with earlier reported findings[13,14]. Increased awareness of dermatological symptoms and signs among health care workers for their presence should provide an opportunity for offering VCT services.

Despite the improved access to ART and improved clinical characteristics of patients attending ART clinics the pattern of clinical presentation has not changed much with time because still patients present late with severe immunosuppression to the HIV clinics. There is therefore a need to improve awareness in the population regarding the availability of care and treatment services, improve HIV counseling and testing so that HIV infected individuals can be identified early in disease progression. This will enhance the appropriate accessibility to preventive and curative services.

We do acknowledge the limitations associated with this retrospective study that did not look at other variables including psycho-social variables that would be associated with access to ARV's. A proper prospective study would provide better information.

The noted limitation with data completeness in some cases has been addressed by ascertaining that the group with complete data was similar sociodemographically with the group that had incomplete data.

Importantly, in our opinion this is the first paper to show that easy access to antiretroviral drugs is associated with improved clinical and immunological characteristics of patients enrolled in HIV clinics.

## Conclusion

Patients enrolled to the MNH HIV clinic were predominantly females, and presented with advanced immune-deficiency. Improved access to HIV care and treatment services seems to be associated with patients' early presentation to the clinics in the course of HIV disease. More efforts need to be put in place to educate the public on the need for regular HIV testing so that the problem is caught earlier, allowing for subsequent appropriate accessibility to preventive and curative services.

## Conflict of interests

The authors declare that they have no competing interests.

## Author's contributions

SFM was involved in acquisition of the data, data analysis and interpretation, and drafting the manuscript. JCM participated in data acquisition, data analysis and drafting the manuscript. JMF was involved in acquiring the data and data analysis and interpretation. SA was involved in critical revision of the manuscript. MB participated in conception and design of the study and data interpretation. EAA was involved in critical revision of the manuscript. ABS participated in data acquisition and critical revision of the manuscript. FMM was involved in the conception and design of the study, data analysis and interpretation and drafting the manuscript. KP contributed in the design and conception of the study and revision of the manuscript. ES was involved in the study conception and design, and critical revision of the manuscript. All authors read and approved the final manuscript.

## Pre-publication history

The pre-publication history for this paper can be accessed here:

http://www.biomedcentral.com/1471-2458/10/291/prepub
